# Availability, pricing, and affordability of antithrombotic medicines in Addis Ababa, Ethiopia: implications for health policy

**DOI:** 10.1186/s40780-025-00408-7

**Published:** 2025-01-09

**Authors:** Selam Birhanu, Melaku Tileku Tamiru, Hanan Muzeyin Kedir, Tamrat Assefa Tadesse

**Affiliations:** Department of Pharmacology and Clinical Pharmacy, School of Pharmacy, College of Health Science, Addis Ababa University, Addis Ababa, Ethiopia

**Keywords:** Antithrombotic medicines, Price, Availability, Affordability, Addis Ababa, Ethiopia

## Abstract

**Background:**

Antithrombotic medications are essential for the management of abnormal clot formation. However, their availability, pricing, and affordability in Ethiopia, particularly in Addis Ababa, have not been comprehensively studied.

**Methods:**

A cross-sectional study was conducted in Addis Ababa, Ethiopia to assess the availability, pricing, and affordability of essential antithrombotic medicines. This study utilized the World Health Organization (WHO) and International Health Action Organization methodology. Five public hospital outpatient pharmacies, four private hospitals, ten private pharmacies, four Kenema pharmacies, and two Red Cross pharmacies in Addis Ababa, Ethiopia, were included in the study. All essential antithrombotic medicines in the 6^th^ edition of Ethiopia’s Essential Medicines List were included in this study. Data were collected for originator brands and the lowest-priced generic medicines available at each medicine outlet.

**Results:**

The availability of low-priced generic (LPG) antithrombotic medicines was 31%, with private hospitals having the highest availability (52%). Original-brand antithrombotic medicines were rarely available, averaging only 3%, with private pharmacies showing a slightly higher availability (10%). The median price of LPG antithrombotic medicines is higher in private settings. Original-brand (OB) antithrombotic medicines in private hospitals and pharmacies were unaffordable, costing between 256.14 and 3,418 days of wages.

**Conclusion:**

The availability of most antithrombotic medicines was low across all sectors compared with the WHO target. Private hospitals showed relatively higher availability of LPG medicines than other pharmacy outlets included in the study. There is a significant disparity between the availability and affordability of LPG and OB medicines. To address these issues, the national drug procurement and distribution systems must be strengthened. Exploring local production and financial assistance programs, implementing effective stock management, regulating medicine prices, promoting high-quality generic medicines, and conducting further research to understand the national landscape are all essential.

## Introduction

Thrombosis, characterized by the formation of blood clots within blood vessels, represents a significant medical condition that poses severe health risks such as stroke, myocardial infarction, and pulmonary embolism [1]. Globally, non-communicable diseases, including those resulting from thrombotic events, contribute substantially to mortality rates, underscoring the critical need for effective treatment strategies [2]. Antithrombotic medicines, including anticoagulants, antiplatelet agents, and thrombolytic drugs, are pivotal in preventing and managing thrombotic disorders by inhibiting clot formation or dissolving existing clots [3].

In Ethiopia, these antithrombotic medicines are considered integral to the country’s Essential Medicines List, which outlines the medications necessary to address the population’s healthcare needs [4]. However, accessibility and affordability of essential medicines remain significant challenges, particularly in low-income countries such as Ethiopia, where healthcare infrastructure limitations, economic disparities, and supply chain inefficiencies often restrict access to vital medicines [5, 6]. Recent studies have highlighted the profound impact of accessible healthcare services and affordable medicines on improving health outcomes, prompting increased investment in healthcare infrastructure, and policy reforms in Ethiopia and other similar settings [7]. The WHO’s ambitious goal of achieving universal health coverage by 2030 emphasizes equitable access to essential medicines through sustainable pricing and procurement strategies tailored to national contexts [8]. However, challenges such as high medicine prices persist, posing substantial barriers to access in many countries and necessitating comprehensive assessments to inform evidence-based policies and interventions [9].

The availability, pricing, and affordability of medicines are critical determinants of universal access to health care. Global studies, including those conducted in Ethiopia, have identified significant shortages of essential medicines such as antithrombotic agents, leading to suboptimal treatment outcomes and higher healthcare costs [6, 10]. Given the significant disparities in access to healthcare in Ethiopia, it is crucial to evaluate the availability, pricing, and affordability of antithrombotic medicines to identify barriers and opportunities for improving the local healthcare system.

Access to affordable essential medicines remains a critical global issue, particularly in low-and middle-income countries, where access barriers disproportionately affect vulnerable populations [6]. Despite efforts to incorporate essential medicines into national healthcare policies in Ethiopia, significant gaps remain in ensuring consistent availability and affordability throughout the healthcare system [11]. Previous studies have highlighted deficiencies in medicine supply chains, regulatory frameworks, and pricing strategies that exacerbate disparities in access to healthcare [9]. Effective management of thrombotic disorders requires timely access to appropriate medications; however, disparities in availability and affordability persist [12]. In Addis Ababa, disparities in medicine availability between the public and private sectors have been documented, reflecting broader challenges in healthcare service delivery and resource allocation [11]. High medicine prices, influenced by factors such as import costs, taxes, and markups, further restrict access to essential antithrombotic medicines, particularly in private healthcare settings [8].

Comprehensive assessments of medicine availability, pricing, and affordability are essential for evidence-based policy decisions to achieve universal health coverage and improve health outcomes [13]. By evaluating the status of antithrombotic medicines, this study sought to offer insights that could help improve medical access and affordability, thereby contributing to sustainable healthcare development in Ethiopia and elsewhere. Therefore, this study aimed to assess the availability, pricing, and affordability of antithrombotic medicines in Addis Ababa, Ethiopia, using methodologies adapted from WHO and Health Action International (HAI) [14]. These findings provide critical insights into the current challenges and potential strategies to enhance medicine access, affordability, and equitable distribution, ultimately contributing to improved health outcomes for patients at risk for thrombotic events in Ethiopia.

## Methods

### Study area, design, and period

Addis Ababa, the capital of Ethiopia, is the country’s largest city and primary hub for health care services. The city has a well-developed healthcare system, with over 50 public and private hospitals. In addition, it has more than 100 public health centers, several hundred pharmacies (operated by licensed pharmacists) throughout the city, and numerous drug stores (operated by licensed pharmacy technicians) that provide essential medical supplies and services to residents. Drug stores generally have a more limited range of medicines and a narrower scope of practice and usually do not handle most antithrombotic medications. Therefore, they were excluded from the study. A cross-sectional survey was conducted to collect data on the availability, pricing, and affordability of antithrombotic medication. Data were collected from April 1 to 30, 2024.

### Health facilities/medicine outlets selection

We selected public health facilities that have been providing anticoagulation management services for at least one year and stock essential medicines for anticoagulation management. Based on the WHO/HAI standardized sampling methodology to assess one region in a country, at least five public hospital outpatient pharmacy outlets and nearby medicine outlets from other types of pharmacies were selected [14]. Using this approach, we included a total of 25 medicine outlets that consisted of five public hospitals, four private hospitals, ten private pharmacies, four Kenema Pharmacies, and two Red Cross Pharmacies. Kenema Pharmacy is a network of non-profit community pharmacies managed by the Addis Ababa Health Bureau in Ethiopia. As of May 2023, Kenema Pharmacy had 43 branches across the city, making it one of the largest public pharmacy networks in Addis Ababa. These pharmacies are integral to the healthcare system and provide services, such as prescription processing, medication dispensing, and health promotion. The mission of Kenema Pharmacy is to ensure the availability of essential medicines and prioritize affordability and accessibility to meet the diverse needs of the population.

### Study medicines selection

Fifteen essential antithrombotic medicines were selected based on the Ethiopian Essential Medicines List 6^th^ edition [15]. For each medicine, two product types were surveyed: originator brand (OB), referring to the brand-name proprietary product, and low-priced generic (LPG), the most affordable generic equivalent available in each pharmacy [14].

### Data collection and quality assurance

A structured questionnaire, adapted from the WHO/HAI standard methodology, was used to gather data on the price, availability, and affordability of antithrombotic medicines. The WHO/HAI methodology was chosen for its standardized approach, enabling global comparability and consistent benchmarking of medicine availability, pricing, and affordability across countries. This framework places the findings within a global context and highlights trends and disparities to guide targeted interventions [14]. This study included all antithrombotic medicines listed on the 6th Essential Medicine List of Ethiopia. Data were collected for both the OB and LPG and were available at each outlet. Two final-year pharmacy students collected data under the supervision of the investigator to ensure accuracy and resolve any incomplete or incorrect entries. A pre-test was conducted in a private pharmacy to confirm the clarity and suitability of the tool. Data quality was further ensured through random supervisor checks and a double-entry system, and discrepancies were verified using the workbook data checker function.

### Data management and analysis

Data were entered into the automated WHO/HAI Medicine Price and Availability Workbook, a customized Microsoft Excel (V 15.0) tool designed for data entry and analysis. After entry, the workbook automatically generated summary tables, which formed the basis of further analysis. Before data collection, the list of medicines to be surveyed was input into the workbook to finalize and generate the medicine price data collection form. All collected data were carefully reviewed and recorded in an Excel spreadsheet and the median price ratios and interquartile ranges for each medicine were calculated.

### Availability

The availability of each studied medicine was measured by its physical presence in the medicine outlets according to its specified strength and dosage form on the data collection date for both the LPG and originator brand options at each pharmacy outlet. Medicine availability was calculated as the percentage of outlets where a specific medicine was in stock on the survey day at each included medicine outlet divided by the number of pharmacy outlets visited. Percentage availability was calculated as follows:

$$\begin{gathered}\%\,availability\,of\,the\,medicine = \hfill \\\,\frac{{number\,of\,pharmacy\,outlets\,having\,the\,medicine}}{{number\,of\,pharmacy\,outlets\,inspected}}{\times100} \hfill \\ \end{gathered}$$

### Price

Medicine prices were obtained from pharmacy price lists or local prices written on the medicine packaging. These local prices were converted to United States dollars (USD) using the exchange rate on the first day of data collection (1 USD = 56.95 ETB). The median price ratio (MPR) is calculated as follows:

$$MPR = \,\frac{{Median\,unit\,price\,of\,the\,medicine\,in\,US\textdollar}}{{Internation\,reference\,price\,in\,US\textdollar}}$$

An MPR greater than 1 indicates that local prices are higher than the international reference prices.

### Affordability

Affordability was determined by dividing the cost of a one-month supply of medicines by the wages of the lowest-paid government worker to buy a specific antithrombotic medicine for one course of therapy.

$$\begin{gathered}Affordability\,calculated\,as = \hfill \\\,\,\,\,\frac{{Price\,of\,medicine\,for\,one\,month}}{{daily\,wage\,of\,the\,lowest\,paid\,government\,worker}} \hfill \\ \end{gathered}$$

A result below one indicated that the medicine was affordable, whereas a result above one indicated that it was unaffordable.

### Ethical considerations

Ethical clearance was obtained from the Ethical Review Committee of the School of Pharmacy Addis Ababa University, Ethiopia (ERB/SOP/579/16/2024). Additionally, permission to access the study facilities was secured from the Addis Ababa Health Bureau (14/14/16073/227). Informed verbal consent was obtained from the managers or heads of each medicine outlet to collect relevant data on the availability, price, and affordability of antithrombotic medicines.

## Results

### Availability of LPG antithrombotic medicines

The mean availability of LPG antithrombotic medicines was 31% across all the surveyed healthcare sectors. Red Cross pharmacies had the lowest availability of antithrombotic medicines (20%), whereas private hospitals had the highest availability (52%). Warfarin 5 mg tablet, clopidogrel 75 mg tablet, and acetylsalicylic acid 81 mg were the most available antithrombotic medicines with availability rates of 91%, 67%, and 65%, respectively. However, enoxaparin injections (20 mg/0.2 ml and 40 mg/0.4 ml) and alteplase powder (10 mg) were unavailable from all surveyed outlets during the study period (Table 1).

**Table 1:** Percentage availability of LPG medicines in Addis Ababa, Ethiopia

Medicine name, strength, and dosage form	Public Hospitals (*n* = 5)	Private Hospitals (*n* = 4)	Private Pharmacies (*n* = 10)	Red Cross Pharmacies (*n* = 2)	Kenema Pharmacies (*n* = 4)	Average	
Warfarin 5 mg tablet	100	75	80	100	100%	91	
Unfractionated heparin 5000U/ml injection	60	100	70	0	50	56	
Enoxaparin 20 mg/0.2 ml injection	0	0	0	0	0	0	
Enoxaparin 40 mg/0.4 ml injection	0	0	0	0	0	0	
Enoxaparin 60 mg/0.6 ml injection	80	0	0	0	50	26	
Rivaroxaban 10 mg tablet	0	75	20	25	50	34	
Rivaroxaban 15 mg tablet	0	75	10	0	0	17	
Rivaroxaban 20 mg tablet	0	75	20	0	0	19	
Acetylsalicylic acid 81 mg tablet	100	50	50	75	50	65	
Acetylsalicylic acid 100 mg tablet	0	75	60	50	100	57	
Clopidogrel 75 mg tablet	60	75	50	50	100	67	
Alteplase 10 mg powder for injection,	0	0	0	0	0	0	
Streptokinase 1.5 million IU powder for injection	0	25	10	0	0	7	
Apixaban 2.5 mg tablet	0	75	0	0	0	15	
Apixaban 5 mg tablet	0	75	0	0	0	15	
Mean availability	27	52	25	20	33	31	

### Availability of originator-brand antithrombotic medicines

The overall availability of originator-brand antithrombotic medicines is extremely low, with an average of 3% across all sectors. However, private pharmacies and hospitals had availability (10% and 5%, respectively) and none of the public health facilities had OB antithrombotic medicines in their stock. Enoxaparin injections (20 mg/0.2 ml, 40 mg/0.4 ml, and 60 mg/0.6 ml) and rivaroxaban tablets (10 mg and 15 mg) were the only originator brand medicines found in private medicine outlets, with overall mean availability rates of 5%, 17%, and 16% for enoxaparin and 4% for both dosages of rivaroxaban in the study health facilities (Table 2).

**Table 2:** Percentage availability of originator brand of antithrombotic medicines in Addis Ababa, Ethiopia

Medicine name, strength, and dosage form	Public hospitals (*n* = 5)	Private hospitals (*n* = 4)	Private pharmacies (*n* = 10)	Red Cross pharmacies (*n* = 2)	Kenema Pharmacies (*n* = 4)	Average
Warfarin 5 mg tablet	0	0	0	0	0	0
Unfractionated heparin 5000U/ml injection	0	0	0	0	0	0
Enoxaparin 20 mg/0.2 ml injection	0	25	0	0	0	5
Enoxaparin 40 mg/0.4 ml injection	0	75	10	0	0	17
Enoxaparin 60 mg/0.6 ml injection	0	50	30	0	0	16
Rivaroxaban 10 mg tablet	0	0	20	0	0	4
Rivaroxaban 15 mg tablet	0	0	20	0	0	4
Rivaroxaban 20 mg tablet	0	0	0	0	0	0
Acetylsalicylic acid 81 mg tablet	0	0	0	0	0	0
Acetylsalicylic acid 100 mg tablet	0	0	0	0	0	0
Clopidogrel 75 mg tablet	0	0	0	0	0	0
Alteplase 10 mg powder for injection,	0	0	0	0	0	0
Streptokinase 1.5 million IU Powder for injection	0	0	0	0	0	0
Apixaban 2.5 mg tablet	0	0	0	0	0	0
Apixaban 5 mg tablet	0	0	0	0	0	0
Overall	0	5	10	0	0	3

### Price of antithrombotic medicines

All LPG of antithrombotic medicines analyzed were found to be available in both public and private retail pharmacies, and higher median buyer prices were found in private pharmacies than in public hospitals. The median price ratio (MPR) of LPG antithrombotic medicines was 1.39, 11.85, 10.4, 6.08, and 2.0, in public hospitals, private pharmacies, and 2.25 in Kenema pharmacies, respectively (Table 3). The details of the MPR, including the minimum and maximum values as well as the 25th and 75th percentiles, are provided in Table 3. Because there were insufficient health facilities to store OB antithrombotic medicines during the study period, the MPR for originator brand medicines was not calculated.

**Table 3:** Median price ratio of LPGs antithrombotic medicines in Addis Ababa, Ethiopia

Medicine name, strength, and dosage form	Lowest price MPR values				
	Public hospitals (*n* = 5)	Private hospitals (*n* = 4)	Private pharmacies (*n* = 10)	Red Cross Pharmacies (*n* = 2)	Kenema pharmacies (*n* = 4)
Warfarin 5 mg tablet	2.62	1.59	3.14	3.14	3.15
Unfractionated heparin 5000U/ml injection	23	55.7	54.48	NA	39.38
Enoxaparin 20 mg/0.2 ml injection	NA	NA	NA	NA	NA
Enoxaparin 40 mg/0.4 ml injection	NA	NA	10.7	NA	NA
Enoxaparin 60 mg/0.6 ml injection	1.39	NA	4.02	NA	1.08
Acetylsalicylic acid 81 mg tablet	0.95	9	13	1	1.35
Acetylsalicylic acid 100 mg tablet	NA	48.23	100.43	38.30	NA
Clopidogrel 75 mg tablet	0.38	11.80	51.87	9.02	0.34
Streptokinase 1.5 million IU powder for injection	NA	2.73	2.05	NA	NA
Median MPR	1.39	10.4	11.85	6.08	2.25
Max MPR	23	55.7	100.43	38.3	83.88
Min MPR	0.38	1.59	2.05	1	0.34
25th percentile	0.665	2.445	3.36	1.535	0.895
75th percentile	12.81	50.098	53.8275	30.98	38.78

NA: not applicable

### Affordability of LPG antithrombotic medicines

Most LPG antithrombotic medicines require a significant number of days’ wages for the lowest-paid government employees to purchase standard treatment. For example, enoxaparin 60 mg/0.6 ml injection was found to require a 1022.62 days’ wage in public hospitals, making it an unaffordable medicine (Table 4).

**Table 4:** Number of days of wages of the lowest-paid government worker needed to purchase standard treatments of LPG antithrombotic medicines in Addis Ababa, Ethiopia

Medicine name, strength, and dosage form	Medicine outlet type				
	Public hospitals	Private hospitals	Private pharmacies	Red Cross pharmacies	Kenema pharmacies
Warfarin 5 mg tablet DDD = 7.5 mg	7.05	4.30	8.96	8.96	8.96
Unfractionated 5000U/ml heparin DDD = 10 TU	760.4	1834.69	1792.14	NA	1295.41
Enoxaparin 20 mg/0.2 ml injection	NA	NA	NA	NA	NA
Enoxaparin 40 mg/0.4 ml injection	NA	NA	NA	NA	NA
Enoxaparin 60 mg/0.6 ml injection	1022.62	NA	NA	NA	792.71
Rivaroxaban 10 mg tablet	NA	42.38	24.54	48.69	44.16
Rivaroxaban 15 mg tablet	NA	111.8	153.51	NA	NA
Rivaroxaban 20 mg tablet	NA	103.10	66.57	NA	NA
Apixaban 2.5 mg tablet	NA	58.91	NA	NA	NA
Apixaban 5 mg tablet	NA	45.82	NA	NA	NA
Clopidogrel 75 mg tablet	1.80	55.52	42.44	55.02	1.62
Streptokinase 1.5 million IU powder for injection	NA	4909.98	3682.48	NA	NA

NA: not applicable; DDD: defined daily dose

All OB antithrombotic medicines found in private hospitals and pharmacies were unaffordable, costing between 256.14- and 3418-day wages (Table 5).

**Table 5:** Number of days of wages of the lowest-paid government worker needed to purchase standard treatments of OB in Addis Ababa, Ethiopia

Medicine name	Affordability				
	Public hospitals	Private hospitals	Private pharmacies	Red Cross pharmacies	Kenema pharmacies
Enoxaparin Injection 20 mg/0.2 ml	NA	1104	NA	NA	NA
Enoxaparin Injection 40 mg/0.4 ml	NA	1743	2373.15	NA	NA
Enoxaparin Injection 60 mg/0.6 ml	NA	3418	2945.99	NA	NA
Rivaroxaban Tablet, 10 mg DDD = 20 mg	NA	NA	423.48	NA	NA
Rivaroxaban Tablet, 15 mg DDD = 20 mg	NA	NA	334.45	NA	NA
Rivaroxaban Tablet, 20 mg DDD = 20 mg	NA	NA	256.14	NA	NA

NA: not applicable ; DDD: defined daily dose

## Discussion

The availability and affordability of essential medicines are critical to ensuring adequate access to healthcare, particularly in resource-limited settings, such as Ethiopia. Improving these factors is one of the most important objectives of the national health policy. Essential medicines are intended to be available at all times within functioning healthcare systems in adequate amounts, in appropriate dosage forms, with assured quality, and at a price that individuals and communities can afford. However, this ideal is far from reality in many parts of the world, including Ethiopia, where the poor availability and high cost of essential medicines remain major public health problems [16].

This study examined the availability, pricing, and affordability of nine essential antithrombotic medicines across various healthcare facilities in Addis Ababa, Ethiopia, and highlighted significant challenges. Despite Addis Ababa being the country’s capital and hub for major hospitals that serve patients across the nation, this study revealed a lack of availability of these critical medicines. The overall availability of LPG antithrombotic medicines was only 31%, which is significantly less than the WHO target of 80% availability of necessary medicines to treat non-communicable diseases [17]. This low availability is particularly concerning given the city’s role as a referral center for cardiac health services. The situation is even more alarming for OBs, with an overall availability of only 3%, indicating a heavy reliance on generic alternatives. The absence of key medicines, such as enoxaparin injections and alteplase powder for injection, from all surveyed outlets underscores the critical need to address supply chain bottlenecks and improve access to specialized medicines. While the lack of availability of brand products was noted, it is important to acknowledge that this does not necessarily indicate a lack of access to the molecule itself. In many cases, a wider availability of generic forms may sufficiently meet the population’s needs. Our inclusion of brand availability aimed to provide additional context for the discussion of medication accessibility by recognizing that the WHO methodology typically focuses on generic availability.

The findings of this study are consistent with those from other low- and middle-income countries where similar challenges in medicine availability have been reported. For example, a study conducted in Eastern Ethiopia found that only 46.97% of essential cardiovascular medicines were available, highlighting systemic issues in the procurement and distribution of these drugs [18]. In contrast, studies in developed countries often report significantly higher availability rates, typically exceeding 90%, reflecting more robust healthcare systems and better resource allocation [19]. This stark contrast between high- and low-income countries underscores the persistent global health disparities and the need for targeted interventions in resource-poor settings. Furthermore, the impact of formularies and related price-negotiation power in developed countries may play a critical role in ensuring higher medicine availability.

The study also revealed significant disparities in the pricing of antithrombotic medicines between public and private sectors. The median price ratios (MPRs) for LPG antithrombotic medicines were substantially higher in private hospitals than in public hospitals, reflecting potential price gouging in the private sector. For instance, the MPR for warfarin 5 mg tablets was 3.14 in private pharmacies, compared to 2.62 in public hospitals. This price difference may contribute to the inaccessibility of these medicines to many patients, particularly to those with limited financial resources. Such disparities are not unique to Ethiopia and similar patterns have been observed in other low-income countries where the private sector often dominates medicine pricing, leading to affordability issues [20].

Affordability is a major concern, as demonstrated in this study. Many antithrombotic medicines require a significant number of days’ wages for the lowest-paid government employees to purchase a standard course of treatment, making them inaccessible to the majority of the population. For example, enoxaparin 60 mg/0.6 ml injection required a 1022.62 days’ wage in public hospitals, which is far beyond the reach of most individuals. This aligns with prior findings in similar settings, where the cost of essential medicines often places an unbearable financial burden on the patients. For instance, in Malawi, the budget impact of adopting newer oral anticoagulants for stroke prevention in atrial fibrillation is significant, raising concerns regarding the sustainability of such treatments in resource-constrained environments [21].

By contrast, developed countries have mechanisms in place to mitigate the financial burden of medicines on patients, including government subsidies, comprehensive insurance coverage, and well-regulated pricing policies. For example, in countries such as Canada and the United Kingdom, essential medicines are often covered under national healthcare schemes, ensuring that patients have access to them with little or no out-of-pocket expenses. This highlights the need for similar policy interventions in Ethiopia, such as expanding insurance coverage, regulating medicine prices, and encouraging the use of high-quality generics to improve affordability [22, 23].

The findings of this study highlight the broader global trend of disparities in the availability and affordability of medicines. While structural challenges such as weak supply chains, inadequate regulatory frameworks, and economic constraints continue to impede access to essential medicines in low- and middle-income countries [24], developed countries benefit from more robust healthcare systems that ensure higher availability and better affordability [25]. However, even within developed countries, access to medicines can be uneven, particularly among marginalized populations. This suggests that, while economic status is a critical factor, healthcare system design, insurance coverage, and regulatory policies also play crucial roles in determining access to essential medicines.

### Strengths and limitations of the study

This study examined the availability, pricing, and affordability of antithrombotic medicines in Addis Ababa, Ethiopia, highlighting critical issues that impact patient outcomes and healthcare policy. This study’s comprehensive approach covers various healthcare sectors, including public hospitals, private pharmacies, and specialized outlets, using WHO/HAI methodology to ensure reliable and globally comparable data. It explores both LPG and OB medicines, offering a nuanced view of the market dynamics and access challenges. However, this study’s findings are limited in scope because they focus solely on Addis Ababa, which may not reflect conditions across Ethiopia. Using LPG to calculate the availability of medicines may overlook a broader range of generic options, particularly if the lowest-priced option represents only a small share of the generic market. Future studies could evaluate the availability of all generic options to provide a more comprehensive assessment. In addition, using the lowest-paid government workers’ wages as a benchmark for affordability may not fully capture the economic reality of the broader population. The absence of international reference prices for some medicines also limits the affordability analysis. Despite these limitations, this study provides valuable insights into policy interventions aimed at improving access to essential medicines in Ethiopia. Expanding research to cover more regions and using broader affordability benchmarks would enhance understanding and inform better healthcare strategies.

## Conclusion and recommendations

This study examined the availability, pricing, and affordability of antithrombotic medicines in Addis Ababa, Ethiopia to identify the major deficiencies that hinder effective healthcare. Key findings showed that the availability of these essential medicines was significantly below the WHO’s target, especially for the originator brands. Medicines are more expensive in private healthcare facilities than in public facilities, making them unaffordable for many patients. To address these challenges, we recommend enhancing the national drug procurement and distribution system, implementing price regulations, promoting the use of high-quality generic medicines, and expanding health insurance coverage to include essential medicines. It also emphasizes the importance of ongoing research and collaboration among stakeholders, including the government, pharmaceutical companies, healthcare providers, and patient advocacy groups, to ensure equitable access to antithrombotic medicines and advance Ethiopia’s goals for universal health coverage and improved health outcomes.

## Data Availability

Data is available upon request to the corresponding author.

## Abbreviations

USD: United States Dollar
DDD: Defined daily dose
IU: International Unit
LPG: Low-priced generic
MPR: Median price ratio
NA: Not applicable
OB: Originator brand
WHO/HAI: World Health Organization (WHO) and International Health Action Organization
